# Testicular Torsion in the Absence of Severe Pain: Considerations for the Pediatric Surgeon

**DOI:** 10.3390/children8060429

**Published:** 2021-05-21

**Authors:** Alexander Kapp, David Troxler, Friederike Prüfer, Stefan Holland-Cunz, Martina Frech, Stephanie J. Gros

**Affiliations:** 1 Department of Pediatric Surgery, University Children’s Hospital Basel, 4031 Basel, Switzerland; alexanderkapp@hotmail.com (A.K.); Stefan.Holland-Cunz@ukbb.ch (S.H.-C.); martina.frech@ukbb.ch (M.F.); 2 Department of Clinical Research, University of Basel, 4031 Basel, Switzerland; 3 Pediatric Emergency Medicine Unit, University Children’s Hospital Basel, 4031 Basel, Switzerland; david.troxler@ukbb.ch; 4 Department of Radiology, University Children’s Hospital Basel, 4031 Basel, Switzerland; friederike.pruefer@ukbb.ch

**Keywords:** painless testicular torsion, pain-reduced testicular torsion, testicular salvage, testicular ultrasound, bell-clapper deformity

## Abstract

Testicular torsion is a surgical emergency. Early diagnosis and surgical treatment are vital in order to preserve the affected gonad. Current surgical teaching emphasizes sudden, severe, persistent, unilateral scrotal pain as a cardinal symptom of testicular torsion. We present the case of unilateral testicular torsion in a 14-year-old patient who presented with the absence of severe pain. Despite a delayed presentation to the emergency department, the gonad could be salvaged successfully. Literature on the topic of testicular torsion presenting with minimal pain is limited. Nevertheless, pediatric surgeons might be faced with cases similar to the one we describe. Underestimating this phenomenon might lead to a delay of treatment. In such cases, ultrasound can be a beneficial addition in the diagnosis and accelerate definitive operative treatment. The presented case clearly demonstrates that, although we do not include testicular torsion without severe pain in our surgical teaching algorithms, we might encounter it in our clinical practice.

## 1. Introduction

Testicular torsion is a surgical emergency. Early diagnosis and surgical treatment are vital in order to preserve the affected gonad. The majority of cases occur during childhood and puberty, and commonly associated symptoms include sudden, severe pain and vegetative symptoms [1,2]. The incidence of testicular torsion has two peaks, one in neonates (pre- or perinatal testicular torsion) and one during puberty [3]. The estimated overall incidence ranges from 1:4000 in the general male population to 3.8:100,000 in males under 18 years of age [4,5]. Neonatal torsions usually occur extravaginally and have a poor salvage rate of around 9%, while teenage torsions typically occur intravaginally and are associated with a significantly higher salvage rate [6,7]. One reason for this might be the ability of older children to better communicate their symptoms. Most torsions cannot be related to any particular event, although 4–10% are diagnosed following a testicular trauma [3,8,9]. The bell-clapper deformity is characterized by a complete enclosing of the testis, epididymis and the distal part of spermatic cord by the tunica vaginalis and predisposes for intravaginal testicular torsion [10]. The familial inheritance of this deformity is a subject of discussion [11].

The majority of textbooks and scientific articles define testicular torsion as being characterized by one or more of the following symptoms or clinical signs:

- Sudden, severe, persistent, unilateral scrotal pain;
- Vegetative symptoms such as nausea and emesis;
- Visible displacement of the testicle, either in rotation or height;
- Swelling and/or tenderness of the scrotal sac;
- Absence of cremasteric reflex.

These symptoms usually lead to a timely presentation of the primary care physician or to the emergency room, although the majority of patients do not present immediately [1,2]. Possible patient-specific reasons for delayed presentation with any of these symptoms may be fear, embarrassment among pubertal children, reduced pain sensitivity, or insufficient communication abilities, especially in very young or neurological patients. External reasons for delayed diagnosis or treatment include deficient health system infrastructure, lack of clinical experience, or misjudgment of the condition by the examining health professionals. Further diagnostics supporting the differential diagnosis include ultrasound with duplex sonography, urine analysis, blood analysis and tumor markers.

Differential diagnoses of scrotal pain and swelling in children include:

- Testicular torsion;
- Torsion of appendix testis;
- Hydrocele;
- Varicocele;
- Scrotal hernia;
- Epididymitis/orchitis;
- Tumor;
- Trauma.

Detorsion of the testicle should be achieved within 6–8 h in order to avoid the necrosis of the testicle [12]. One recent review article supports this claim through an analysis of testicle survival time, showing that documented testicle survival gradually decreases from 97.2%, if testicular detorsion is achieved within 6 h of the onset, to only 24.4% between 25 and 48 h and below 10% after 48 h [13].

Testicular torsion is a surgical diagnosis, if it manifests with the above-described symptoms. Current surgical teaching emphasizes sudden, severe, persistent, unilateral scrotal pain as a cardinal symptom of testicular torsion. We present a case of an oligosymptomatic patient with testicular torsion, which reminds us to consider testicular torsion in the absence of this cardinal symptom.

## 2. Case Presentation

A 14-year-old patient presented to our interdisciplinary pediatric emergency room with scrotal discomfort of 3–5 by the numeric rating system (NRS) and the swelling of the right testicle. These symptoms were first noticed two days prior to presentation but had never been perceived as particularly severe by the patient. No initial point of onset could be identified. Recent trauma or strenuous activities were plausibly denied by the patient. Fever, nausea, emesis or other vegetative symptoms were not described. Sexual activity was negated. The patient did not appear to be shy or embarrassed about the symptoms or the physical examination.

On inspection, the right scrotum was enlarged and showed slight redness but no significantly increased local temperature. Clinical examination revealed an enlarged and slightly elevated right testicle compared to the left testicle. The testicle was neither tender nor painful on palpation. Prehn sign was negative. The cremasteric reflexes were regular on both sides. The value of commonly known symptoms as it is emphasized in surgical teaching is set against the actual symptoms of the patient in Table 1.

**Figure 1:**
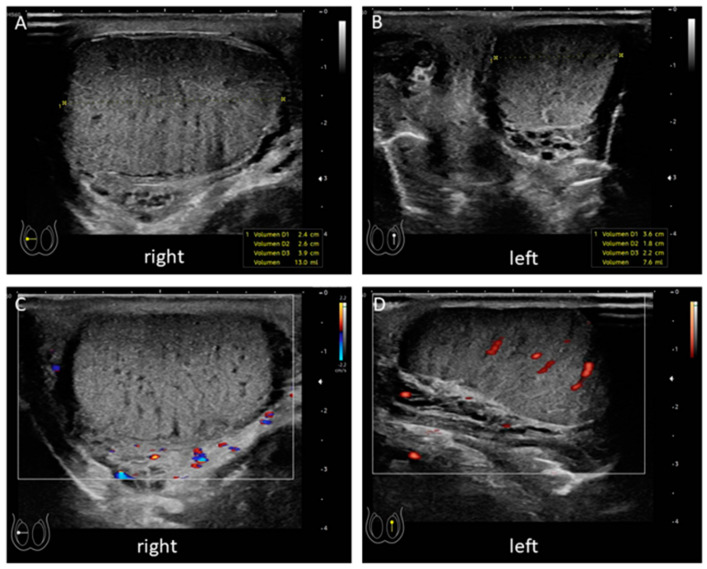
Point of care ultrasound. The initial ultrasound in the pediatric emergency room showed and enlarged the right testicle (**A**) compared to the left side (**B**). No perfusion of the right testicle could be detected (**C**) while the left testicle showed signs of central perfusion (**D**).

As severe scrotal pain was absent, laboratory tests and ultrasound were performed and revealed a slightly elevated C-reactive protein. Point-of-care ultrasound in the emergency room showed a right testicle with an increased volume of 13 mL versus 7.6 mL contralaterally. There were no clear signs of perfusion on the right side (Figure 1).

**Figure 2:**
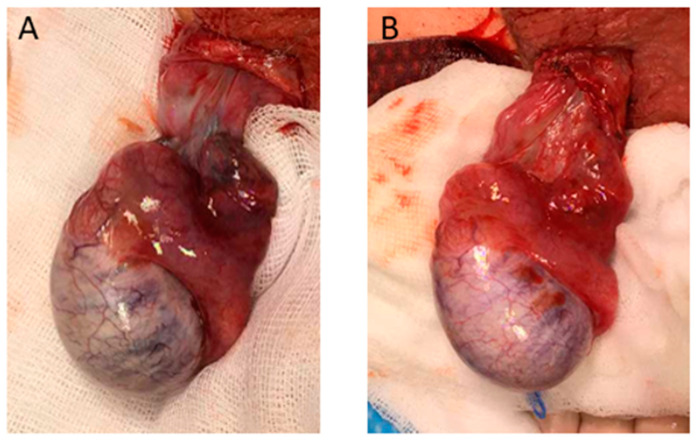
Intraoperative findings. Upon surgical exploration of the testicle, a 540° torsion was resolved. (**A**) shows the testicle immediately after detorsion; (**B**) 20 min after detorsion testicle had recovered sufficiently to attempt orchidopexy.

Thus, immediate exploratory surgery via a scrotal approach was initiated. Intraoperatively we found a 540° torsion of the right testicle with hypoperfusion and significant static swelling of the testicular vessels, as well as a bell-clapper deformity. After detorsion, the testicle had recovered sufficiently after 20 min in warm towels for the decision to be made to perform orchidopexy (Figure 2). This was followed by an orchidopexy of the left testicle, which also showed a bell-clapper deformity.

**Figure 3:**
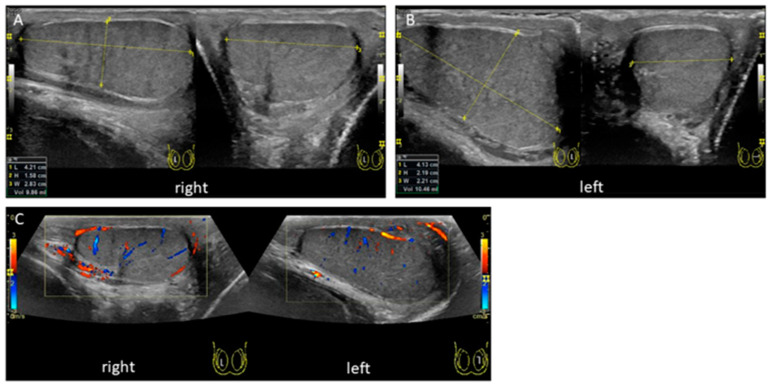
Ultrasonography 8 weeks after detorsion. An ultrasound was performed 8 weeks after the operation. The right testicle recovered its parenchymal structure and size (**A**). Left testicle is shown in (**B**). Regular central perfusion was observed equally on both sides (**C**).

The patient could be discharged the following day and the follow-up examination 8 weeks after the operation showed completely healed wounds and an excellent testicular parenchymal structure and perfusion, as shown in the ultrasound performed by a pediatric radiologist (Figure 3). Interestingly, the patient did not perceive the postoperative pain as severe or even very uncomfortable.

During the hospital stay and upon follow-up, the history was taken regarding pain perception and neurological deficits. The patient had not previously noticed unexplained wounds or hematomas, or difficulties in temperature sensing. He had not experienced lack of balance or motion steadiness. According to him and his family, he had been eating a balanced diet with appropriate portions for his age. The neurological examination showed no signs of neuropathy, including inconspicuous reaction to painful stimuli, normal reflexes, muscle profile, vibration sensitivity and no deformity of the feet.

The case was discussed with the pediatric neurologist and neither history nor clinical examination warranted any further diagnostic procedures such as EMNG at this time. However, we advised the family and pediatrician to be attentive to new symptoms and in such a case advised for consecutive early reevaluation.

Written consent was given by the patient and his guardian to use clinical information as presented in accordance with the institutional consent form as approved by the Ethikkommission Nordwest- und Zentralschweiz (EKNZ).

## 3. Discussion

Testicular torsion is a surgical and clinical diagnosis. In the literature as well as in medical and surgical textbooks, it is described as associated with sudden, intense, unrelenting pain, often resulting in vegetative symptoms, the combination of which should prompt the patient to seek medical support. Our case presentation clearly shows that this is not always the case. The patient was plausibly and objectively not in great distress. His discomfort levels were, even taking interpersonal differences in pain perception into consideration, mild to moderate. His main complaint was the scrotal swelling more than the pain. While clinical presentations asked for further investigations to check the differential diagnoses, the ultrasound findings did not. This case raises several questions for the pediatric surgeon to consider. Is this just an exceptional case? Is the patient’s pain perception compromised? Why do we not teach the possibility of a nearly painless testicular torsion? What is the value of ultrasound for diagnosing testicular torsion?

Notably, an article by Lyon in 1961 reported that “the classic symptoms … may often not be the first sign of torsion” [14]. The author supported this claim with reports of seven pediatric patients above the age of one with slight (*n* = 3), moderate (*n* = 2) or even absent pain (*n* = 2) [14]. Among these patients, none had severe swelling of the scrotum and in six cases, an ipsilateral bell-clapper deformity was found. He deducted that pain accompanying the scrotal swelling may not be necessary and concluded that this standard set of symptoms should be questioned in favor of an early diagnosis. Apart from this report, the painless or pain-reduced testicular torsion was not described in the literature. Taking Lyon’s report into account, the phenomenon we describe here might not be so rare but might oftentimes just not be reported.

The primary diagnosis of testicular torsion can be made clinically in the presence of severe classical symptoms as described above, ideally more than one. Studies have shown that a classical symptom such as an absent cremasteric reflex has a high sensitivity of up to 99%, while a normal cremasteric reflex does not rule out torsion [2,3]. A study evaluating the diagnosis of scrotal disorders point out that clinical judgement may be impaired if pain and swelling prevent a thorough palpation of the testicle [15]. Additionally, when classical symptoms appear inconsistently, pain is intermittent and non-specific symptoms occur, diagnosis and treatment may be delayed [11]. Two common scores for evaluating the probability of testicular torsion—TWIST (testicular workup for ischemia and suspected torsion) and BAL (Boettcher alert score)—have shown high sensitivity and specificity for predicting testicular torsion [11,16,17,18]. However, they rely on classical clinical symptoms (pain, nausea/emesis, displacement of the testicle, abnormal cremasteric reflex, swelling and hardened testicle), the majority of which were not present in our case.

Several imaging methods are available for assessing the differential diagnoses of testicular torsion in pediatric patients. Mainly, ultrasound might be beneficial in the acute situation. Ultrasound can be used as a tool to complement the clinical impression and to distinguish differential diagnoses [19]. In a study on 33 surgically confirmed cases of testicular torsion, the sensitivity of color doppler ultrasonography was found to be 85% and that of high-resolution ultrasonography even higher with 94%, reaching 100% when combining both methods [20]. The specificity of duplex sonography in pubertal and prepubertal boys has been reported as being comparable in both age groups, suggesting that this method is applicable in both age groups [21].

Although ultrasound is easily accessible and not harmful due to the lack of ionizing radiation, its quality is highly dependent on the examiners’ experience as well as the sophistication of technical equipment [11,19]. Multiple factors such as small testicle volume or incomplete torsion may lead to misdiagnosis [11]. Ultrasound has been shown to delay treatment only minimally while having a high sensitivity of 88.9% in the diagnosis of testicular torsion [2,22]. Additional procedures such as computed tomography (CT), magnetic resonance imaging (MRI) and nuclear imaging hardly play a role in acute cases.

In the case of a questionable diagnosis and an inconclusive ultrasound, immediate exploratory surgery should be performed. Surgery is therefore still considered the gold standard for diagnosing testicular torsion [23]. Pain relief as a temporary measure may be possible by manual detorsion. However, it should be followed by subsequent semi-elective surgical fixation [2,3,11]. When performing surgical fixation, a bilateral approach should be considered, as the predisposing bell-clapper deformity occurs bilaterally in 66-100% [3,10]. The incidence of this deformity ranges between 12 and 16% [10,24]. Although recent literature on this topic is scarce, prophylactic contralateral fixation has not been shown to increase morbidity and is therefore recommended [1,2,4,11,14,25].

In the presented case, we have no clinical reason to suspect an underlying pathology of pain perception. Subjective pain experience in healthy children may vary according to multiple factors such as sex, age, and coping mechanisms [26]. Studies suggest that pain tolerance increases with age, while sensitivity towards and perceived duration of pain are higher in children than in adults [27,28]. A high pain threshold, however, may mask the severity of the underlying condition. Additionally, rare diseases such as congenital insensitivity to pain should be kept in mind when dealing with unusually high pain tolerance [29]. As a further differential diagnosis neuropathy should be considered.

## 4. Conclusions

Taken together, the literature on the topic of pain-reduced testicular torsion is limited. Nevertheless, pediatric surgeons might be faced by cases similar to the one we describe. Clinically, there is no explanation for the absence of severe pain in this particular patient. Underestimating the possibility of painless testicular torsion might lead to a delay of treatment. In such cases, ultrasound can be an important beneficial addition in the diagnosis and accelerate definitive operative treatment. The presented case clearly demonstrates that, although we do not include testicular torsion without severe pain in our surgical teaching algorithms, we might encounter it in our clinical practice.

## Figures and Tables

**Table 1:** Symptoms of testicular torsion.

Commonly Known	Stressed in Surgical Teaching	Present Is the Presented Case
Sudden, severe, persistent, unilateral scrotal pain	+++	−
Vegetative symptoms such as nausea and emesis	++	−
Visible displacement of the testicle, either in rotation or height	+	+
Swelling of the scrotal sac	+	+
Tenderness of the scrotal sac	+	−
Absence of cremasteric reflex	++	−

## Data Availability

Not applicable.
